# Association between the *LIPG* polymorphisms and serum lipid levels in the Maonan and Han populations

**DOI:** 10.1002/jgm.3071

**Published:** 2019-02-04

**Authors:** Shuo Yang, Rui‐Xing Yin, Liu Miao, Qing‐Hui Zhang, Yong‐Gang Zhou, Jie Wu

**Affiliations:** ^1^ Department of Cardiology, Institute of Cardiovascular Diseases, The First Affiliated Hospital Guangxi Medical University Nanning Guangxi China

**Keywords:** endothelial lipase, environmental factors, lipids, single nucleotide polymorphism

## Abstract

**Introduction:**

The Maonan population is a relatively isolated minority in China. Little is known about endothelial lipase gene (*LIPG*) single nucleotide polymorphisms (SNPs) and serum lipid levels in the Chinese populations. The present study aimed to detect the association of several *LIPG* SNPs and environmental factors with serum lipid levels in the Chinese Maonan and Han populations.

**Methods:**

In total, 773 subjects of Maonan ethnicity and 710 participants of Han ethnicity were randomly selected from our previous stratified randomized samples. Genotypes of the *LIPG* rs2156552, rs4939883 and rs7241918 SNPs were determined by polymerase chain reaction‐restriction fragment length polymorphism, and then confirmed by direct sequencing.

**Results:**

The allelic (rs2156552, rs4939883 and rs7241918) and genotypic (rs2156552 and rs4939883) frequencies were different between the two ethnic groups (*p* < 0.05–0.01). The minor allele carriers had lower apolipoprotein (Apo)A1/ApoB ratio (rs2156552 and rs7241918), high‐density lipoprotein cholesterol (HDL‐C) and apolipoprotein (Apo)A1 (rs2156552) levels and higher ApoB levels (rs4939883) in the Han population, and lower HDL‐C (rs2156552, rs4939883 and rs7241918) levels in the Maonan minority than the minor allele non‐carriers (*p* < 0.0167 after Bonferroni correction). Subgroup analyses according to sex showed that the minor allele carriers had a lower ApoA1/ApoB ratio (rs2156552 and rs7241918) and higher ApoB levels (rs7241918) in Han males, and lower ApoA1 and HDL‐C levels in Maonan females than the minor allele non‐carriers (*p* < 0.0167–0.001).

**Conclusions:**

The present study demonstrates the association between the *LIPG* polymorphsims and serum lipid levels in the two ethnic groups. These associations might have an ethnic‐ and or/sex‐specificity.

## 1 INTRODUCTION

Coronary artery disease (CAD) is the most common cause of mortality, morbidity and a major contributor to the financial burden in developing countries.^1^ Dyslipidemia, particularly hypertriglyceridemia and hypercholesterolemia, is a well‐described independent predictor for atherosclerosis and CAD. Low‐density lipoprotein cholesterol (LDL‐C) has been considered to be the major lipid risk factor and main target of lipid‐lowering therapy in most national guidelines. Previous studies have shown a highly consistent, inverse correlation between plasma concentrations of high‐density lipoprotein cholesterol (HDL‐C) and its major protein apolipoprotein (Apo)A1 and atherosclerotic cardiovascular disease risk in humans.^2^ In addition, clinical data have indicated that each 1% increase in the serum concentration of HDL‐C can decrease cardiovascular risk by 2–3%.^3^ Although previous research reported that the risk for dyslipidemia is largely attributed to an unhealthy lifestyle, such as poor nutrition,^4^ lack of exercise,^5^ excessive drinking and smoking,^6^ there is now strong evidence suggesting that predisposition to the development of lipid disorders begins with heredity.^7^ The heritability of serum HDL‐C levels in the Strong Heart Family study and HERITAGE family study has been estimated at 50% and 52%, respectively.^8^, ^9^ By contrast, the associated variants in genome‐wide association studies (GWAS) accounted for only 5–8% of the variation in the HDL‐C levels.^10^ However, the specific genetic variants that contribute to serum HDL‐C levels in the diverse ethnic groups are largely unknown.

The endothelial lipase gene (*LIPG*, as known as EL, EDL, PRO719; Gene ID: 9388; HGNC ID:6623) is the most recent member assigned to the triglycerde (TG) lipase family, which was reported to play a physiological role in the modulation of HDL‐C metabolism.^11^ Serum HDL‐C levels are regulated in part by the lipase enzyme family, and its members include lipase, lipoprotein lipase (LPL) and hepatic lipase (HL).^12^ LIPG is highly homologous to LPL and HL, both of which are critical to the metabolism of lipids carried on plasma lipoproteins.^13^ A study of the lipolytic activity showed that *LIPG* has more phospholipase activity and relatively less TG lipase activity and can hydrolyze HDL phospholipids *ex vivo*.^14^ Another study demonstrated that overexpression of *LIPG* in mice liver by adenovirus‐mediated gene transfer results in a remarkable decrease in HDL‐C and ApoA1 levels.^15^ Antibody inhibition studies in wild‐type and *LIPG* knockout mice demonstrated that inhibition of *LIPG* causes siginificantly increased HDL‐C levels.^16^ Vergeer *et al*.^17^ showed that *LIPG* uses its phospholipase activity to hydrolyze HDL‐C (its primary substrate) in a dose‐dependent manner. Additionly, a previous study reported that, although the preferred substrate of LIPG is HDL, LIPG is still capable of hydrolyzing apoB‐containing lipoproteins [very LDL (VLDL)/lDL)].^18^ Indeed, Broedl *et al*.^19^ demonstrated that LIPG can reduces the levels of serum VLDL‐C and LDL‐C in atherosclerosis‐prone mouse models. These data suggest that, in addition to its role in HDL metabolism, LIPG may also contribute to VLDL and LDL metabolism. These loss‐of function experiments suggest that *LIPG* could be a physiological regulator of lipid metabolism. Despite the obvious functional evidence for an influence of *LIPG* on altered serum lipid levels in animal models, it remains to be determined whether this receptor has an equally important function in humans.

The human *LIPG* is located on chromosome 18q21.1 and is expressed in a variety tissues, including the liver, placenta, lung and testis.^20^ Several SNPs in the *LIPG* have been found to be associated with serum HDL‐C concentrations in some studies but not in others.^21^, ^22^, ^23^, ^24^, ^25^, ^26^, ^27^, ^28^, ^29^ The main reason for inconsistency in serum lipid levels among these studies may be the different ethnic, genetic, sex, health and environmental factors and their interactions. Therefore, further research will be necessary to characterize the full impact of these SNPs on lipid metabolism in different racial and ethnic groups.

China is a multi‐ethnic country with 56 ethnic groups, and the Maonan ethnicity is a minority in South China that possesses a unique and colourful traditional culture. According to China's sixth national census in 2010, the Maonan population size is about 107 166 (Rank 37) and most individuals live in the Huanjiang Maonan Autonomous County in Guangxi Zhuang Autonomous Region. As a result of their special customs and culture, including conservative intra‐ethnic marriages, dietary habits and lifestyle, we speculate that some hereditary characteristics and genotypes of serum lipid metabolism‐realted genes in this population might be different from those of local Han ethnic group. In addition, to the best of our knowledge, the association of *LIPG* SNPs and serum lipid levels has not been reported previously in the Maonan population. Thus, the present study aimed to assess the association of *LIPG* (rs2156552, rs4939883 and rs7241918) SNPs and several environmental factors with serum lipid concentrations in the Maonan and Han populations.

## 2 MATERIALS AND METHODS

### 2.1 Subjects

The participants in the present study included 710 unrelated individuals of Maonan ethnicity (267 males, 37.61%; 443 females, 62.39%) and 773 unrelated participants of Han ethnicity (306 males, 39.59%; 467 females, 60.41%). They were randomly selected from our previous stratified randomized samples. Three generations of the Maonan and Han participants were living in Guangxi Huanjiang Maonan Autonomous County (see Supporting information, Figure S1) and all participants were agricultural workers. The age of the participants ranged from 25 to 80 years, with a mean ± SD age of 56.05 ± 11.67  and 57.14 ± 14.99 years in the Han and Maonan populations (*p* > 0.05), respectively. All study subjects were essentially healthy, with no history of cardiovascular disease such as CAD and stroke, diabetes, hyper‐ or hypothyroids, and chronic renal disease. We excluded subjects who had a history of taking medications known to affect serum lipid levels (lipid‐lowering drugs such as statins or fibrates, beta‐blockers, diuretics, or hormones) before the blood sample was drawn. The present study was approved by the Ethics Committee of the First Affiliated Hospital, Guangxi Medical University (No: Lunshen‐2014‐KY‐Guoji‐001; 7 March 2014). Informed consent was taken from all participants after they received a full explanation of the study. All procedures of the investigation were carried out accordance with the Declaration of Helsinki.

### 2.2 Epidemiological survey

The survey was carried out using internationally standardized methods, in accordance with a common protocol.^30^ Information on demographics, socioeconomic status and lifestyle factors was collected with standardized questionnaires. The alcohol information included questions about the number of liangs (approximately 50 g) of rice wine, corn wine, rum, beer or liquor consumed during the preceding 12 months. Alcohol consumption was categorized into groups of grams of alcohol per day: ≤ 25 and > 25. Smoking status was categorized into groups of cigarettes per day: ≤ 20 and > 20. Several parameters, such as blood pressure, height, weight, waist circumference and body mass index (BMI), were measured or calculated. The methods of measuring above parameters have been described in a previous study.^31^

### 2.3 Biochemical measurements

A fasting venous blood sample of 5 ml was drawn from the participants. A part of the sample (2 ml) was collected into glass tubes and used to determine serum lipid levels, and another part (3 ml) was shifted to tubes with anticoagulants (4.80 g/l citric acid, 14.70 g/l glucose and 13.20 g/l tri‐sodium citrate) and used to extract DNA. Measurements of serum total cholesterol (TC), TG, HDL‐C and LDL‐C levels in the samples were performed via enzymatic methods using commercially available kits (RANDOX Laboratories Ltd, Crumlin, UK; Daiichi Pure Chemicals Co., Ltd, Tokyo, Japan). Serum ApoA1 and ApoB levels were measured by the immunoturbidimetric immunoassay using a commercial kit (RANDOX Laboratories Ltd). All determinations were performed with an auto‐analyzer (Type 7170A; Hitachi Ltd, Tokyo, Japan) in the Clinical Science Experiment Center of the First Affiliated Hospital, Guangxi Medical University.^32^

### 2.4 DNA amplification and genotyping

Genomic DNA of the samples was extracted from peripheral blood leucocytes in accordance with the phenol–chloroform method.^33^ The extracted DNA was stored at 4°C until analysis. Genotyping of the *LIPG* SNPs was performed using the polymerase chain reaction and restriction fragment length polymorphism (PCR‐RFLP). The sequences of the forward and backward primers, restriction enzymes used and the size of the restriction fragments are provided in the Supporting information (Table S1). Each 25 μl of the PCR reaction mixture consisted of 2.0 μl of genomic DNA, 1.0 μl of each primer (10 μmol/l), 12.5 μl of 2 × *Taq* PCR Master mix (constituent: 0.1 U *Taq* polymerase/μl, 500 μmol/l dNTP each and PCR buffer) and 8.5 μl of ddH_2_O (DNase/RNase‐free). PCR was performed with an initialization step of 95°C for 5 min, followed by 30 s denaturing at 95°C, 30 s of annealing at 60°C and 30 s of elongation at 72°C for 33 cycles. The amplification was completed by a final extension at 72°C for 7 min. Following electrophoresis on a 2.0% agarose gel with 0.5 μg/ml ethidium bromide, the amplification products were visualized under ultraviolet light. Subsequently, each restriction enzyme reaction was performed with 5.0 μl of amplified DNA, 8.8 μl of nuclease‐free water, 1.0 μl of 10 × buffer solution and 0.2 μl of restriction enzymes in a total volume of 15 μl and digested at 37°C overnight. After restriction enzyme digestion of the amplified DNA, genotypes were identified by electrophoresis on 2% ethidium‐bromide stained agarose gels and visualized with ultraviolet illumination. Genotypes were scored by an experienced reader who was blinded to the epidemiological and serum lipid results.

### 2.5 DNA sequencing

Eighteen samples (each genotype in two) detected by the PCR‐RFLP were also confirmed by direct sequencing. The PCR products were purified by low melting point gel electrophoresis and phenol extraction, and then the DNA sequences were analyzed using an ABI Prism 3100 (Applied Biosystems, Foster City, CA, USA) at Shanghai Sangon Biological Engineering Technology & Services Co., Ltd., People's Republic of China.

### 2.6 Diagnostic criteria

The normal values of serum TC, TG, HDL‐C, LDL‐C, ApoA1, ApoB levels and the ApoA1/ApoB ratio in our Clinical Science Experiment Center were 3.10–5.17, 0.56–1.70, 1.16–1.42 and 2.70–3.10 mmol/l, 1.20–1.60 and 0.80–1.05 g/l, and 1.00–2.50, respectively. The individuals with TC > 5.17 mmol/l and/or TG > 1.70 mmol/l were defined as hyperlipidemic.^34^ The hypertension diagnosis standard was in accordance with the criteria of the 1999 and 2003 World Health Organization‐International Society of Hypertension Guidelines for the management of hypertension.^35^ The diagnostic criteria for being overweight and obesity were in accordance with the Cooperative Meta analysis Group of China Obesity Task Force. Normal weight, overweight and obesity were defined as a BMI of < 24, 24–28 and > 28 kg/m^2^, respectively.^36^

### 2.7 Statistical analysis

Epidemiological data were recorded on a pre‐designed form and managed with Excel (Microsoft Corp., Redmond, WA, USA). Data analysis was performed using SPSS, version 22.0 (IBM Corp., Armonk, NY, USA). The quantitative variables are presented as the mean ± SD (serum TG levels are presented as medians and interquartile ranges). Allele frequency was determined via direct counting and the Hardy–Weinberg equilibrium was verified with a standard goodness‐of‐fit test. Genotype distribution between the two groups was analyzed by the chi‐squared test. General characteristics between the two ethnic groups were compared using Student's unpaired *t*‐test. The association between genotypes and serum lipid parameters was tested by analysis of covariance (ANCOVA). Any SNPs associated with the lipid profiles at *p* < 0.0167 (corresponding to *p* < 0.05 after adjusting for three independent tests with Bonferroni correction) were considered statistically significant. Sex, age, BMI, blood pressure, alcohol consumption and cigarette smoking were adjusted for the statistical analysis. Multivariable linear regression analyses with stepwise modeling were used to determine the correlation between the genotypes (rs2156552: AT/TT = 0, AA = 1; rs4939883: CT/TT = 0, CC = 1; rs7241918: GT/TT = 0, GG = 1) and several environmental factors with serum lipid levels in a combined population of Maonan and Han, Maonan, Han, males and females; respectively. *p* < 0.05 (two‐sided) was considered statistically significant.

## 3 RESULTS

### 3.1 General characteristics and serum lipid profiles

General characteristics and serum lipid parameters for the Han and Maonan populations are summarized in Table 1. Levels with respect to systolic blood pressure, diastolic blood pressure, pulse pressure, waist circumference and the percentages of subjects who consumed alcohol and smoked cigarettes were lower in the Han than in the Maonan population (*p* < 0.05–0.001). The levels of serum HDL‐C and the ratio of ApoA1 to ApoB were higher in the Han than in the Maonan population, whereas the levels of ApoB were lower in the Han than in the Maonan population (*p* < 0.05–0.001). There were no significant differences with respect to sex ratio, age structure, height, weight, BMI, glucose, serum TC, TG, LDL‐C and ApoA1 levels between the two ethnic groups (*p* > 0.05 for all).

**Table 1:** Comparison of demographic, lifestyle characteristics and serum lipid levels between the Han and Maonan populations

Parameter	Han	Maonan	*t* (*x* ^2^)	*p*
Number	710	773		
Male/female	267/443	306/467	0.612	0.434
Age (years)	56.03 ± 11.62	57.14 ± 13.99	1.381	0.164
Height (cm)	153.98 ± 7.73	153.80 ± 8.08	0.412	0.681
Weight (kg)	52.90 ± 8.84	53.16 ± 10.63	−0.482	0.630
Body mass index (kg/m^2^)	22.28 ± 3.25	22.37 ± 3.58	−0.437	0.662
Waist circumference (cm)	75.10 ± 7.80	76.75 ± 9.22	−3.425	0.001
Smoking status, *n* (%)				
Non‐smoker	583 (82.11)	605 (78.27)		
≤ 20 cigarettes/day	104 (14.65)	150 (19.40)		
> 20 cigarettes/day	23 (3.24)	18 (2.33)	6.684	0.035
Alcohol consumption, *n* (%)				
Non‐drinker	612 (86.20)	605 (78.27)		
≤ 25 g/day	46 (6.48)	94 (12.16)		
> 25 g/day	52 (7.32)	74 (9.57)	17.694	0.000
Systolic blood pressure (mmHg)	129.02 ± 19.53	135.53 ± 23.79	−5.303	0.000
Diastolic blood pressure (mmHg)	81.21 ± 11.52	83.07 ± 12.29	−2.738	0.006
Pulse pressure (mmHg)	47.82 ± 15.33	52.47 ± 17.46	−4.982	0.000
Glucose (mmol/l)	6.17 ± 1.85	6.15 ± 1.43	0.248	0.804
Total cholesterol (mmol/l)	4.95 ± 0.94	5.00 ± 1.03	−0.978	0.328
Triglyceride (mmol/l)	1.17 (0.54)	1.30 (0.51)	−2.489	0.013
HDL‐C (mmol/l)	1.75 ± 0.52	1.64 ± 0.38	3.868	0.000
LDL‐C (mmol/l)	2.87 ± 0.77	2.89 ± 0.82	−0.487	0.626
ApoA1 (g/l)	1.38 ± 0.25	1.36 ± 0.27	1.155	0.248
ApoB (g/l)	0.82 ± 0.17	0.88 ± 0.20	−5.194	0.000
ApoA1/ApoB	1.72 ± 0.47	1.66 ± 0.50	2.108	0.035

HDL‐C, high‐density lipoprotein cholesterol; LDL‐C, low‐density lipoprotein cholesterol; Apo, apolipoprotein. The value of triglyceride is presented as the median (interquartile range), the difference between the two ethnic groups was determined by the Wilcoxon Mann–Whitney test.

### 3.2 Genotypic and allelic frequencies

**Figure 1:**
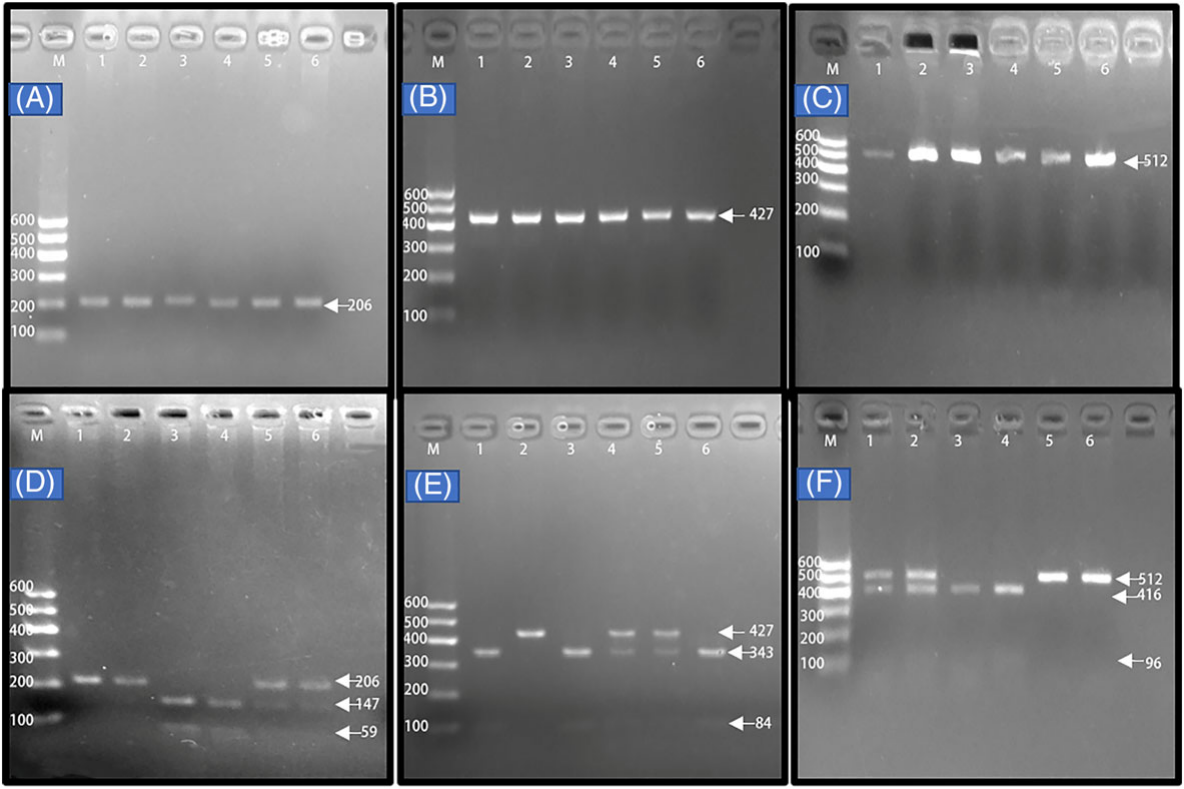
The PCR products of the samples and the genotypes of the *LIPG* SNPs. Lane M is the 100‐bp marker ladder; (A to C) lanes 1–6 are the PCR products: A, rs2156552 SNP (206 bp); B, rs4939883 SNP (427 bp); C, rs7241918 SNP (512 bp); D, genotyping of the rs2156552 SNP: lanes 1 and 2, TT genotype (206 bp); lanes 3 and 4, AA genotype (147 and 59 bp); and lanes 5 and 6, AT genotype (206, 147 and 59 bp); E, genotyping of the rs4939883 SNP: lanes 1, 3 and 6, CC genotype (343 and 84 bp); lanes 2, TT genotype (427 bp); and lanes 4 and 5, CT genotype (427, 343 and 84 bp); F, genotyping of the rs7241918 SNP: lanes 1 and 2, GT genotype (512‐, 416‐ and 96‐bp); lanes 3 and 4, GG genotype (416 and 96 bp); and lanes 5 and 6, TT genotype (512 bp)

**Figure 2:**
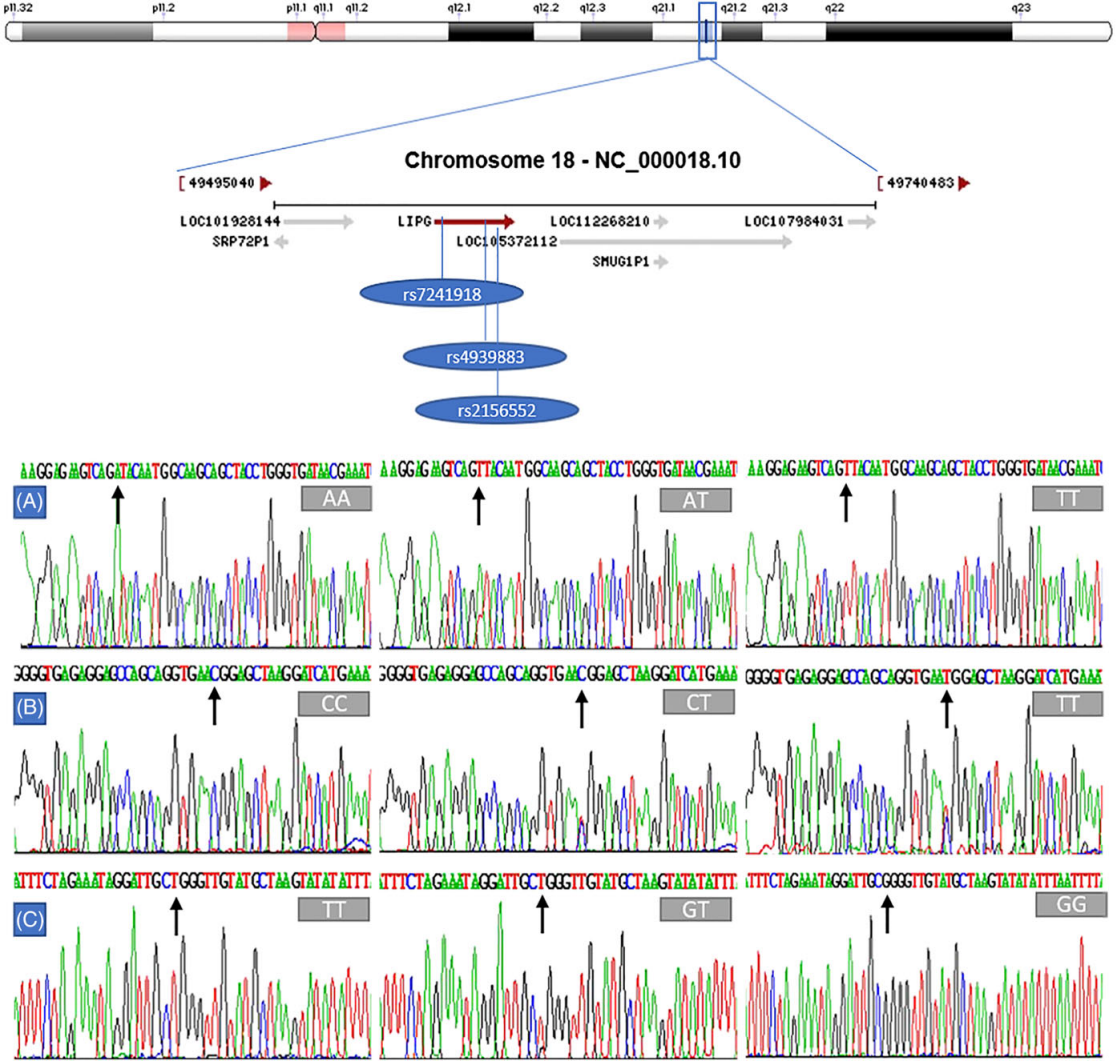
A part of the nucleotide sequences of the *LIPG* SNPs. A, rs2156552 SNP; B, rs4939883 SNP; C, rs7241918 SNP

**Figure 3:**
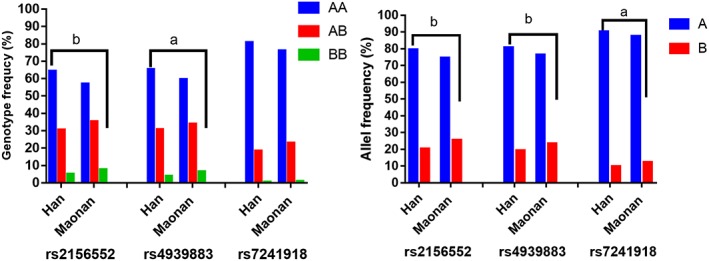
The genotypic and allelic frequencies of the *LIPG* SNPs in the Maonan and Han populations. Allele A: rs2156552A, rs4939883C, or rs7241918T; allele B: rs2156552T, rs4939883T, or rs7241918G; genotype AA: rs2156552AA, rs4939883CC, or rs7241918TT; genotype AB: rs2156552AT, rs4939883CT, or rs7241918GT; genotype BB: rs2156552TT, rs4939883TT, or rs7241918GG. ^a^ *p* < 0.05; ^b^ *p* < 0.01; *p*_HWE_ > 0.05 for all

**Figure 4:**
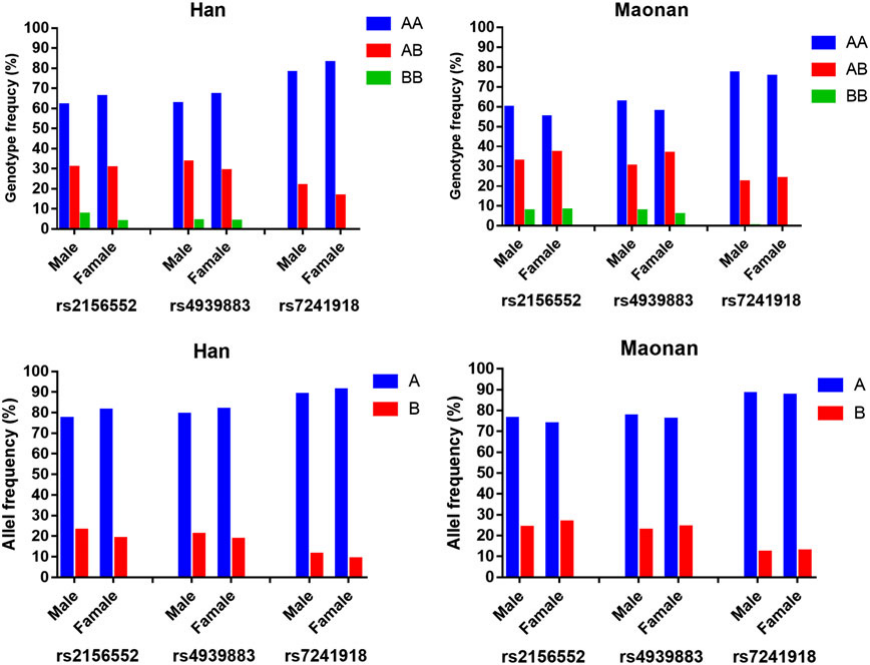
The genotypic and allelic frequencies of the *LIPG* SNPs between males (M) and famales (F) in the Maonan and Han populations. Allele A: rs2156552A, rs4939883C, or rs7241918T; allele B: rs2156552T, rs4939883T, or rs7241918G; genotype AA: rs2156552AA, rs4939883CC, or rs7241918TT; genotype AB: rs2156552AT, rs4939883CT, or rs7241918GT; genotype BB: rs2156552TT, rs4939883TT, or rs7241918GG. ^a^ *p* < 0.05; ^b^ *p* < 0.01; *p*_HWE_ > 0.05 for all

The PCR products of the samples and the results of genotyping of the *LIPG* SNPs are shown in Figure 1. The genotypes detected by PCR‐RFLP were also confirmed by direct sequencing (Figure 2). As shown in Figure 3, the genotypic and allelic frequencies of the *LIPG* rs2156552 and rs4939883 SNPs were different between the two ethnic groups. The rs2156552T and rs4939883T allele frequencies and the rs2156552AT/TT and rs4939883CT/TT genotype frequencies were higher in the Maonan than in the Han population (*p* < 0.05–0.01). The allelic frequencies of the *LIPG* rs7241918 SNP were also different between the two ethnic groups. The rs7241918G allele frequency was higher in the Maonan than in the Han population (*p* < 0.05). There was no significant difference with respect to either genotypic or allelic frequencies between males and females of both ethnic groups (Figure 4).

### 3.3 Genotypes and serum lipid levels

**Figure 5:**
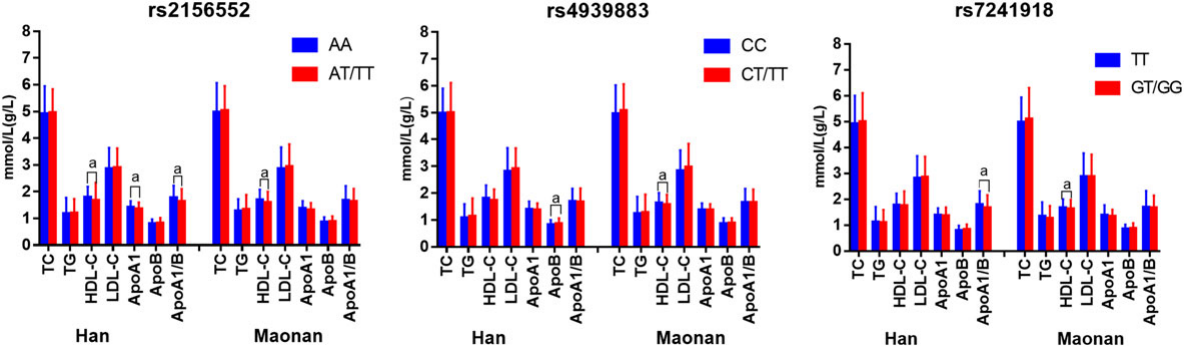
The *LIPG* genotypes and serum lipid levels in the Han and Maonan populations. TC, total cholesterol; TG, triglyceride; HDL‐C, high‐density lipoprotein cholesterol; LDL‐C, low‐density lipoprotein cholesterol; ApoA1, apolipoprotein A1; ApoB, apolipoprotein B; ApoA1/ApoB, the ratio of apolipoprotein A1 to apolipoprotein B. The value of TG is presented as the median (interquartile range). The difference between the genotypes was determined by the Kruskal–Wallis test. The *p*‐value was calculated by ANCOVA, using general linear models, and adjusted for age, sex, body mass index, smoking status, alcohol use, glucose and hypertension; *p* < 0.0167 was considered statistically significant after adjusting by the Bonferroni correction). ^a^ *p* < 0.0167; ^b^ *p* < 0.001

**Figure 6:**
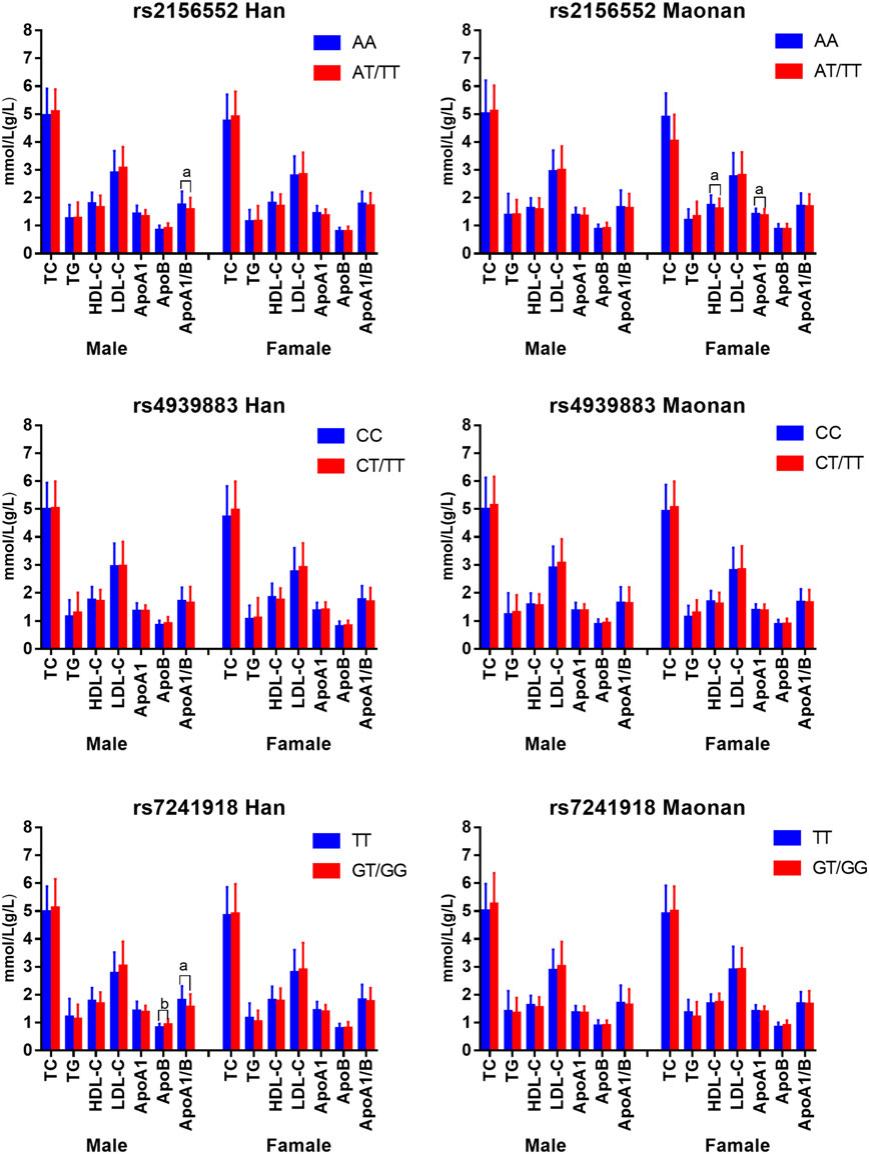
The *LIPG* genotypes and serum lipid levels in males and females of the Han and Maonan populations. TC, total cholesterol; TG, triglyceride; HDL‐C, high‐density lipoprotein cholesterol; LDL‐C, low‐density lipoprotein cholesterol; ApoA1, apolipoprotein A1; ApoB, apolipoprotein B; ApoA1/ApoB, the ratio of apolipoprotein A1 to apolipoprotein B. The value of TG is presented as the median (interquartile range). The difference between the genotypes was determined by the Kruskal–Wallis test. The *p*‐value was calculated by ANCOVA, using general linear models, and adjusted for age, sex, body mass index, smoking status, alcohol use, glucose and hypertension; *p* < 0.0167 was considered statistically significant after adjusting by the Bonferroni correction). ^a^ *p* < 0.0167; ^b^ *p* < 0.001

As shown in Figure 5, the minor allele carriers had a lower ApoA1/ApoB ratio (rs2156552 and rs7241918) and HDL‐C and ApoA1 levels (rs2156552) and higher ApoB levels (rs4939883) in the Han population, and lower HDL‐C (rs2156552, rs4939883 and rs7241918) levels in the Maonan minority than the minor allele non‐carriers (*p* < 0.0167). Subgroup analysis according to sex showed that the minor allele carriers had a lower ApoA1/ApoB ratio (rs2156552 and rs7241918) and higher ApoB levels (rs7241918) in Han males but not females, and lower ApoA1 and HDL‐C levels in Maonan famales but not males than the minor allele non‐carriers (*p* < 0.0167 for all) (Figure 6).

### 3.4 Relative factors for serum lipid parameters

The correlation between genotypes of three SNPs and serum lipid parameters is shown in Table 2. Multivariable linear regression analyses showed that genotypes were associated with HDL‐C levels (rs2156552, rs4939883 and rs7241918) in the Maonan minority, and the ApoA1/ApoB ratio (rs2156552 and rs7241918) and ApoB levels (rs4939883) in the Han population. Serum lipid parameters were also correlated with several environmental factors, such as sex, age, alcohol consumption, cigarette smoking, blood pressure, blood glucose, waist circumference and BMI, in both ethnic groups or in males and females (*p* < 0.05–0.001) (Tables 3 and 4).

**Table 2:** Correlation between the *LIPG* rs2156552, rs4939883 and rs7241918 genotypes and serum lipid levels in the Han and Maonan populations

Lipid	Genotype	B	SE	Beta	*t*	*p*
Han and Maonan						
HDL‐C	Genotype of rs2156552	−0.046	0.022	−0.059	−2.061	0.040
ApoA1	Genotype of rs2156552	0.010	0.004	0.076	2.690	0.007
Han						
ApoA1/ApoB	Genotype of rs2156552	0.114	0.046	0.116	2.513	0.012
	Genotype of rs7241918	−0.142	0.049	−0.135	−2.903	0.004
ApoB	Genotype of rs4939883	−0.095	0.020	−0.225	−4.659	0.000
Maonan						
HDL‐C	Genotype of rs2156552	0.092	0.028	0.118	3.257	0.001
	Genotype of rs7241918	0.065	0.025	0.093	2.574	0.010
	Genotype of rs4939883	0.084	0.029	0.102	2.897	0.004

TC, total cholesterol; TG, triglyceride; HDL‐C, high‐density lipoprotein cholesterol; LDL‐C, low‐density lipoprotein cholesterol; ApoA1, apolipoprotein A1; ApoB, apolipoprotein B; ApoA1/ApoB, the ratio of apolipoprotein A1 to apolipoprotein B; B, unstandardized coefficient; Beta, standardized coefficient.

**Table 3:** Relationship between serum lipid parameters and environmental risk factors in the Han and Maonan populations

Lipid	Risk factor	B	SE	Beta	*t*	*p*
Han and Maonan						
TC	Age	0.009	0.002	0.146	4.160	0.000
	Alcohol consumption	0.107	0.053	0.069	2.032	0.042
	Waist circumference	0.016	0.005	0.136	2.924	0.004
	Diastolic blood pressure	0.005	0.003	0.062	2.023	0.043
TG	Alcohol consumption	0.273	0.082	0.109	3.328	0.001
	Waist circumference	0.045	0.008	0.244	5.439	0.000
	Glucose	0.097	0.029	0.095	3.394	0.001
HDL‐C	Ethnic group	0.104	0.025	0.115	4.093	0.000
	Sex	0.092	0.036	0.101	2.536	0.011
	Age	0.003	0.001	0.092	2.686	0.007
	Cigarette smoking	0.021	0.006	0.094	3.305	0.001
	Alcohol consumption	0.089	0.023	0.129	3.884	0.000
	Waist circumference	−0.009	0.002	−0.171	−3.724	0.000
LDL‐C	Age	0.007	0.002	0.136	3.907	0.000
	Alcohol consumption	−0.134	0.042	−0.108	−3.192	0.001
	Waist circumference	0.014	0.004	0.158	3.387	0.001
ApoA1	Ethnic group	0.105	0.033	0.245	3.187	0.002
	Sex	0.063	0.021	0.120	2.992	0.003
	Cigarette smoking	0.011	0.004	0.083	2.892	0.004
	Alcohol consumption	0.111	0.013	0.280	8.397	0.000
ApoB	Ethnic group	0.035	0.011	0.089	3.265	0.001
	Age	0.001	0.000	0.093	2.788	0.005
	Waist circumference	0.006	0.001	0.261	5.871	0.000
ApoA1/ApoB	Sex	0.102	0.040	0.101	2.589	0.010
	Alcohol consumption	0.147	0.025	0.191	5.888	0.000
	Waist circumference	−0.013	0.003	−0.223	−4.976	0.000
Han						
TC	Age	0.007	0.003	0.120	2.144	0.033
	Waist circumference	0.024	0.010	0.201	2.528	0.012
TG	Waist circumference	0.062	0.011	0.243	5.370	0.000
	Age	−0.012	0.006	−0.093	−1.991	0.047
	Cigarette smoking	0,589	0.159	0.163	3.693	0.000
	Diastolic blood pressure	0.021	0.008	0.126	2.709	0.001
	Glucose	0.128	0.048	0.122	2.676	0.008
HDL‐C	Alcohol consumption	0.099	0.038	0.126	2.595	0.010
	Weight	−0.012	0.003	−0.212	−4.370	0.000
LDL‐C	Age	0.008	0.002	0.160	3.507	0.000
	Waist circumference	0.015	0.004	0.147	3.234	0.001
ApoA1	Alcohol consumption	0.104	0.201	0.254	4.849	0.000
	Weight	−0.007	0.001	−0.220	−4.462	0.000
	Cigarette smoking	0.052	0.025	0.106	2.093	0.037
ApoB	Waist circumference	0.005	0.001	0.222	4.782	0.000
	Alcohol consumption	0.028	0.014	0.107	2.019	0.044
	Height	−0.004	0.001	−0.161	−2.962	0.003
	Sex	−0.053	0.021	−0.151	−2.461	0.014
ApoA1/ApoB	Body mass index	−0.036	0.006	−0.255	−5.682	0.000
Maonan						
TC	Age	0.010	0.003	0.137	3.796	0.000
	Sex	0.192	0.079	0.090	2.426	0.016
	Waist circumference	0.017	0.004	0.153	4.121	0.000
TG	Alcohol consumption	0.320	0.073	0.150	4.389	0.000
	Waist circumference	0.047	0.005	0.317	2.916	0.010
	Glucose	0.085	0.034	0.148	2.491	0.013
HDL‐C	Sex	0.113	0.039	0.145	2.881	0.004
	Age	0.002	0.001	0.089	2.101	0.036
	Cigarette smoking	0.021	0.006	0.135	3.829	0.000
	Alcohol consumption	0.079	0.025	0.132	3.195	0.001
	Waist circumference	−0.011	0.002	−0.261	−4.655	0.000
	Pulse pressure	−0.002	0.001	−0.079	−1.991	0.047
LDL‐C	Age	0.008	0.002	0.149	3.440	0.001
	Alcohol consumption	−0.181	0.055	−0.141	−3.326	0.001
	Waist circumference	0.015	0.005	0.170	2.957	0.003
ApoA1	Sex	0.091	0.025	0.180	3.568	0.000
	Cigarette smoking	0.010	0.004	0.098	2.759	0.006
	Alcohol consumption	0.105	0.016	0.272	6.523	0.000
	Diastolic blood pressure	0.002	0.001	0.087	2.259	0.024
	Pulse pressure	−0.001	0.001	−0.089	−2.224	0.026
ApoB	Age	0.002	0.001	0.142	3.411	0.001
	Waist cirumference	0.006	0.001	0.298	5.442	0.000
ApoA1/ApoB	Alcohol consumption	0.178	0.032	0.225	5.603	0.000
	Age	−0.003	0.001	−0.097	−2.362	0.018
	Waist cirumference	−0.015	0.003	−0.283	−5.197	0.000

TC, total cholesterol; TG, triglyceride; HDL‐C, high‐density lipoprotein cholesterol; LDL‐C, low‐density lipoprotein cholesterol; ApoA1, apolipoprotein A1; ApoB, apolipoprotein B; ApoA1/ApoB, the ratio of apolipoprotein A1 to apolipoprotein B; B, unstandardized coefficient; Beta, standardized coefficient.

**Table 4:** Relationship between serum lipid parameters and environmental risk factors in the males and females of the Han and Maonan populations

Lipid	Risk factor	B	SE	Beta	*t*	*p*
Han/male						
TC	Diastolic blood pressure	0.014	0.006	0.187	2.522	0.013
TG	Waist circumference	0.154	0.047	0.428	3.295	0.001
HDL‐C	Weight	−0.017	0.003	−0.349	−4.926	0.000
	Alcohol consumption	0.096	0.035	0.193	2.720	0.007
LDL‐C	Cigarette smoking	−0.204	0.088	−0.173	−2.319	0.022
ApoA1	Cigarette smoking	0.059	0.029	0.139	2.001	0.047
	Alcohol consumption	0.113	0.023	0.342	4.827	0.000
	Weight	−0.008	0.002	−0.262	−3.781	0.000
ApoB	Body mass index	0.012	0.003	0.241	3.333	0.001
	Glucose	0.015	0.007	0.149	2.081	0.039
ApoA1/ApoB	Alcohol consumption	0.092	0.041	0.161	2.259	0.025
	Body mass index	−0.040	0.009	−0.309	−4.348	0.000
Han/female						
TC	Age	0.008	0.004	0.284	5.011	0.000
	Waist circumference	0.019	0.007	0.144	2.541	0.012
TG	Waist circumference	0.038	0.009	0.243	4.204	0.000
	Diastolic blood pressure	0.016	0.006	0.154	2.675	0.008
	Glucose	0.085	0.033	0.145	2.613	0.009
HDL‐C	Waist circumference	0.010	0.005	0.129	2.196	0.029
LDL‐C	Cigarette smoking	0.204	0.088	0.173	2.319	0.022
ApoA1	Alcohol consumption	0.113	0.023	0.342	4.827	0.000
	Weight	−0.008	0.002	−0.262	−3.781	0.000
	Cigarette smoking	0.059	0.029	0.139	2.001	0.047
ApoB	Body mass index	0.012	0.003	0.241	3.258	0.002
	Glucose	0.015	0.007	0.149	2.081	0.039
ApoA1/ApoB	Alcohol consumption	0.092	0.041	0.161	2.259	0.025
	Body mass index	−0.040	0.009	−0.309	−4.348	0.000
Maonan/male						
TC	Weight	0.023	0.005	0.253	4.465	0.000
TG	Alcohol consumption	0.395	0.126	0.170	3.126	0.002
	Glucose	0.174	0.071	0.144	2.445	0.015
	Waist circumference	0.067	0.011	0.328	6.039	0.000
HDL‐C	Cigarette smoking	0.022	0.005	0.217	4.073	0.000
	Alcohol consumption	0.093	0.025	0.194	3.652	0.000
	Waist circumference	−0.020	0.004	−0.472	−5.328	0.000
	Body mass index	0.026	0.011	0.209	2.385	0.018
LDL‐C	Alcohol consumption	−0.228	0.058	−0.222	−3.939	0.002
	Body mass index	0.046	0.015	0.173	3.078	0.002
ApoA1	Cigarette smoking	0.011	0.004	0.153	2.865	0.004
	Alcohol consumption	0.115	0.018	0.345	5.472	0.000
	Waist circumference	−0.006	0.002	−0.196	−3.681	0.000
ApoB	Weight	0.008	0.001	0.439	7.965	0.000
	Age	0.002	0.001	0.143	2.551	0.011
	Glucose	0.021	0.007	0.156	2.916	0.004
ApoA1/ApoB	Glucose	−0.048	0.020	−0.122	−2.342	0.020
	Alcohol consumption	0.201	0.036	0.290	5.551	0.000
	Waist cirumference	−0.020	0.003	−0.335	−6.430	0.000
Maonan/female						
TC	Age	0.014	0.003	0.190	4.116	0.000
	Waist circumference	0.012	0.006	0.093	2.021	0.044
TG	Waist circumference	0.033	0.004	0.357	4.124	0.000
	Systolic pressure	0.005	0.001	0.145	3.289	0.001
HDL‐C	Waist circumference	−0.010	0.002	−0.223	−4.928	0.000
LDL‐C	Age	0.010	0.002	0.193	4.234	0.000
	Waist circumference	0.016	0.004	0.171	3.753	0.000
ApoA1	Body mass index	−0.007	0.003	−0.114	−2.442	0.015
ApoB	Age	0.002	0.001	0.167	3.453	0.001
	Waist circumference	0.007	0.001	0.298	6.717	0.000
	Systolic pressure	0.001	0.000	0.127	2.563	0.011
ApoA1/ApoB	Waist circumference	−0.016	0.002	−0.307	−6.654	0.000
	Pluse pressure	−0.005	0.001	−0.179	−4.005	0.000
	Height	0.008	0.004	0.102	2.249	0.025

TC, total cholesterol; TG, triglyceride; HDL‐C, high‐density lipoprotein cholesterol; LDL‐C, low‐density lipoprotein cholesterol; ApoA1, apolipoprotein A1; ApoB, apolipoprotein B; ApoA1/ApoB, the ratio of apolipoprotein A1 to apolipoprotein B; B, unstandardized coefficient; Beta, standardized coefficient.

### 3.5 Logistic regression of SNPs and serum lipid levels

As shown in Table 5, the genotype and allelic frequencies of the *LIPG* rs2156552, rs4939883 and rs7241918 SNPs were significantly different in the normal and dyslipidemia groups (*p* < 0.05–0.001).

**Table 5:** The association between the *LIPG* polymorphisms with dyslipidemia in Han and Maonan populations [*n* (%)]

SNP	Genotype	Normal	Dyslipidemia	*x* ^2^	*p*	OR (95% CI)	*p* *
Han		Normal (*n* = 561)	Dyslipidemia (*n* = 149)				
rs2156552	AA	375 (66.84)	82 (55.03)	11.346	0.003	1	
	AT+TT	186 (33.16)	67 (44.97)			1.22 (1.01–1.54)	0.035
	MAF	208 (18.54)	81 (27.18)	12.447	0.002		
rs4939883	CC	383 (68.27)	81 (54.36)	10.058	0.002	1	
	CT + TT	178 (31.73)	68 (45.64)			1.31 (1.09–1.62)	0.0024
	MAF	196 (17.47)	78 (26.17)	15.633	0.0004		
rs7241918	TT	466 (83.07)	109 (73.15)	7.511	0.006	1	
	GT + GG	95 (16.93)	40 (26.85)			1.04 (0.88–1.36)	0.022
	MAF	97 (8.64)	42 (14.09)	12.823	0.002		
Maonan		Normal (*n* = 578)	Dyslipidemia (*n* = 195)				
rs2156552	AA	342 (59.17)	98 (50.26)	4.724	0.030	1	
	AT+TT	236 (40.83)	97 (49.74)			1.41 (1.17–1.76)	0.041
	MAF	272 (23.53)	121 (31.03)	8.591	0.014		
rs4939883	CC	356 (61.59)	104 (53.33)	4.127	0.042	1	
	CT + TT	222 (38.41)	91 (46.67)			1.35 (1.11–1.65)	0.0047
	MAF	258 (22.32)	105 (26.92)	5.745	0.057		
rs7241918	TT	453 (78.37)	135 (69.23)	6.695	0.013	1	
	TG + GG	125 (21.63)	60 (30.77)			1.18 (0.82–1.33)	0.038
	MAF	129 (11.16)	63 (16.15)	10.966	0.004		

MAF, minor allele frequency. *p* defined as chi‐squared test probability; OR, odds ratio; CI, confidence interval.

* *p* defined as logistic test probability.

### 3.6 SNP–environmental interactions on serum lipid levels

The interactions of the *LIPG* SNPs and sex, age, BMI, smoking and drinking on serum lipid levels are shown in Table 6. The rs2156552 SNP interacted with male and cigarette smoking and the rs4939883 and rs7241918 SNPs interacted with alcohol consumption to increase the risk of dyslipidemia in the Han population. Furthermore, in the Maonan population, the rs2156552 SNP interacted with cigarette smoking and hypertension, the rs4939883 SNP interacted with cigarette smoking and diabetes, and the rs7241918 SNP interated with alcohol consumption to increase the risk of lipid disorders.

**Table 6:** The *LIPG* SNPs and hyperlipidemia in the Han and Maonan populations according to stratified risk factors

		Han		Maonan	
Factors	Genotype	OR (95% CI)	*p*	OR (95% CI)	*p*
rs2156552					
Sex					
Male	AA	1		1	
	AT+TT	1.79 (1.22–2.62)	0.003	1.35 (0.75–2.01)	0.359
Female	AA	1		1	
	AT+TT	1.22 (0.88–1.79)	0.308	0.97 (0.63–1.73)	0.867
Age (years)					
≤ 60	AA	1		1	
	AT+TT	0.94 (0.61–1.44)	0.790	0.78 (0.57–1.21)	0.673
> 60	AA	1			
	AT+TT	1.06 (0.69–1.62)	0.067	1.27 (0.93–1.95)	0.261
BMI (kg/m^2^)					
≤ 24	AA	1		1	
	AT+TT	1.22 (0.80–1.89)	0.347	1.13 (0.97–1.89)	0.411
> 24	AA	1		1	
	AT+TT	1.47 (0.79–2.73)	0.217	1.37 (0.87–2.01)	0.188
Smoking					
No	AA	1		1	
	AT+TT	1.05 (0.70–1.55)	0.810	1.19 (0.97–2.07)	0.788
Yes	AA	1		1	
	AT+TT	1.93 (1.21–3.07)	0.001	2.02 (1.80–4.12)	0.007
Drinking					
No	AA	1		1	
	AT+TT	1.26 (0.76–1.98)	0.481	1.13 (0.77–1.94)	0.574
Yes	AA	1		1	
	AT+TT	1.87 (1.06–2.29)	0.053	1.53 (1.14–2.39)	0.421
Hypertension					
No	AA	1		1	
	AT+TT	0.72 (0.554–1.16)	0.729	1.09 (0.91–1.56)	0.611
Yes	AA	1		1	
	AT+TT	1.21 (0.87–1.82)	0.563	1.43 (1.03–2.21)	0.006
Diabetes					
No	AA	1		1	
	AT+TT	1.18 (0.92–1.66)	0.053	1.31 (1.05–2.69)	0.518
Yes	AA	1		1	
	AT+TT	1.36 (0.85–1.86)	0.061	1.61 (1.16–2.71)	0.073
rs4939883					
Sex					
Male	CC	1		1	
	CT + TT	1.11 (0.72–1.60)	0.637	1.17 (0.70–1.89)	0.406
Female	CC	1		1	
	CT + TT	0.89 (0.58–1.39)	0.378	0.93 (0.62–1.40)	0.543
Age (years)					
≤ 60	CC	1		1	
	CT + TT	1.03 (0.72–1.71)	0.541	0.93 (0.72–1.36)	0.616
> 60	CC	1		1	
	CT + TT	1.18 (0.92–2.42)	0.069	1.24 (0.94–2.02)	0.135
BMI (kg/m^2^)					
≤ 24	CC	1		1	
	CT + TT	0.94 (0.65–1.35)	0.745	1.07 (0.72–1.60)	0.411
> 24	CC	1		1	
	CT + TT	1.31 (1.07–2.19)	0.293	1.13 (0.85–1.43)	0.180
Smoking					
No	CC	1		1	
	CT + TT	1.14 (0.83–1.79)	0.554	1.27 (1.06–1.73)	0.061
Yes	CC	1		1	
	CT + TT	1.31 (1.15–2.16)	0.143	1.34 (0.99–2.02)	0.047
Drinking					
No	CC	1		1	
	CT + TT	1.06 (0.81–1.72)	0.462	1.14 (0.88–1.73)	0.263
Yes	CC	1		1	
	CT + TT	1.46 (1.07–2.36)	0.012	1.53 (1.14–2.51)	0.039
Hypertension					
No	CC	1		1	
	CT + TT	0.67 (0.46–1.04)	0.070	1.15 (0.87–1.67)	0.632
Yes	CC	1		1	
	CT + TT	0.92 (0.67–1.27)	0.522	1.23 (0.72–1.92)	0.458
Diabetes					
No	CC	1		1	
	CT + TT	1.21 (0.81–1.92)	0.445	1.13 (0.81–1.79)	0.053
Yes	CC	1		1	
	CT + TT	1.47 (0.91–1.95)	0.205	1.72 (1.35–2.56)	0.001
rs7241918					
Sex					
Male	TT	1		1	
	GT + GG	1.03 (0.80–1.44)	0.339	1.13 (0.81–1.76)	0.062
Female	TT	1		1	
	GT + GG	0.96 (0.65–1.27)	0.553	1.04 (0.74–1.39)	0.770
Age (years)					
≤ 60	TT	1		1	
	GT + GG	0.81 (0.64–1.23)	0.624	1.09 (0.79–1.68)	0.540
> 60	TT	1		1	
	GT + GG	1.12 (0.85–1.47)	0.279	1.17 (0.86–1.67)	0.273
BMI (kg/m^2^)					
≤ 24	TT	1		1	
	GT + GG	0.91 (0.76–1.23)	0.238	1.23 (0.81–1.93)	0.940
> 24	TT	1		1	
	GT + GG	1.16 (0.86–1.74)	0.462	1.35 (0.98–1.89)	0.084
Smoking					
No	TT	1		1	
	GT + GG	1.17 (0.94–1.82)	0.693	0.87 (0.56–1.35)	0.431
Yes	TT	1		1	
	GT + GG	1.57 (1.06–1.95)	0.324	1.15 (0.81–1.69)	0.157
Drinking					
No	TT	1		1	
	GT + GG	1.08 (0.74–1.67)	0.056	1.26 (0.77–1.94)	0.574
Yes	TT	1		1	
	GT + GG	1.35 (1.08–2.19)	0.036	1.53 (1.14–2.39)	0.008
Hypertension					
No	TT	1		1	
	GT + GG	0.86 (0.58–1.26)	0.068	1.09 (0.82–1.64)	0.661
Yes	TT	1		1	
	GT + GG	1.14 (0.93–1.58)	0.403	1.57 (1.14–1.92)	0.060
Diabetes					
No	TT	1		1	
	GT + GG	1.23 (1.05–2.04)	0.104	0.98 (0.61–1.29)	0.430
Yes	TT	1		1	
	GT + GG	1.63 (1.15–2.16)	0.051	1.24 (1.06–2.07)	0.081

OR, odds ratio; CI, confidence interval.

## 4 DISCUSSION

It is well known that circulating levels of blood lipids such as LDL‐C levels are consistently associated with atherosclerosis and the risk of CAD. However, the role of circulationg HDL‐C levels in the development of CAD remains uncertain. In this context, the integration of population genetics and epidemiological studies could help identify targets for new therapies regarding serum lipid management and the prevention of CAD. In the present study, for the first time, we reveal the association of the *LIPG* rs2156552, rs4939883 and rs7241918 SNPs and serum lipid profiles in the Maonan population. Serum TG and ApoB levels were higher, whereas serum HDL‐C levels and the ApoA1/ApoB ratio were lower, in the Maonan than in the Han populations. These results might be owing to differences in lifestyle and genetic factors between the two ethnic groups. The Maonan population still conforms with the custom of consanguineous marriage to cousins of the maternal side (e.g. more than 80% of the Maonan people share the same surname: Tan). Recently, Wang et al. revealed that Maonan people belong to the Southeastern Asian group and are most closely related to the Buyi people,^37^, ^38^ suggesting that the genetic background of the Maonan population may be less heterogeneous within the population. Therefore, we speculate that the hereditary characteristics and some lipid metabolism‐related gene polymorphisms in the Maonan population may be different from those in the Han population.

The genotypic and allelic frequencies of the *LIPG* rs2156552, rs4939883 and rs7241918 SNPs in diverse racial/ethnic groups are not well known. In the present study, we found that the allelic and genotypic frequencies of the rs2156552 and rs4939883 SNPs were different between the Maonan and Han populations. The allele frequencies but not the genotypic frequencies of the rs7241918 SNP were different between the two ethnicities. The T allele frequency of the rs2156552 SNP was lower in the Han than in the Maonan population (20.35% versus 25.42%; *p <* 0.01); the T allele frequency of the rs4939883 SNP was lower in the Han than in the Maonan population (19.30% versus 23.48%; *p* < 0.01); and the G allele frequency of the rs7241918 SNP was also lower in the Han than in the Maonan population (9.79% versus 12.42%; *p* < 0.05); respectively. Sex subgroup analysis showed that there were no conspicuous differences in the genotypic and allelic frequencies of the three SNPs between males and females in the Maonan and Han populations. According to the International 1000 Genomes data‐base (https://www.ncbi.nlm.nih.gov/variation/tools/1000genomes), the minor rs2156552T, rs4939883T and rs7241918G allele frequencies were 19.03%, 19.49% and 16.67% in Europeans, respectively. All of the above minor allele frequencies in European ancestries of the three selected SNPs were significantly different from the Han and Maonan populations. These results reveal that the rare homozygote genotype and allele frequencies of the rs2156552, rs4939883 and rs7241918 SNPs shared a racial/ethnic specificity.

In the last decade, several HDL‐C candidate genes have been identified via association studies and one of the candidate genes found to be associated in GWAS is *LIPG*. Studies using genetically modified mice have suggested that *LIPG* activity negatively influences plasma HDL‐C levels.^16^, ^17^ The high‐level overexpression of *LIPG* in the liver significantly decreased the levels of serum HDL‐C and ApoA1.^17^ A relevant study showed that the *LIPG* mutations are involved in increased monocyte adhesion and uptake in the vessel wall, which is used to indicate the early inflammation step of atherosclerosis. Because a reduced plasma HDL‐C level is a well‐documented and modifiable risk factor for atherosclerotic diseases, including CAD and ischemic stroke, many genetic association studies have investigated the effects of common sequence variants in *LIPG* on HDL‐C levels and diseases related to HDL‐C levels. However, previous findings regarding the association of these SNPs with changes in serum lipid levels are inconsistent. The *LIPG* rs2156552 SNP has been associated with HDL‐C in many nationalities.^23^, ^24^, ^25^ A large Diabetes Genetic Initiative GWAS reported that subjects with the minor allele of rs2156552 had lower serum HDL‐C concentrations than subjects with the major allele (*p* = 2 × 10^−7^).^22^ Another GWAS conducted in 17 723 participants of white European descent also confirmed that the T allele carriers of rs2156552 in *LIPG* were associated with lower levels of HDL‐C (*p* = 1.7 × 10^−12^).^23^ Dumitrescu *et al*.^24^ replicated the association of rs2156552 with plasma HDL‐C levels in diverse populations and found that the minor allele carriers had a negative correlation with HDL‐C in European Americans but an opposite effect in African Americans and American Indians. By contrast, Chung *et al*.^39^ reported that the rs2156552 SNP was associated with the serum HDL‐C level in a Korean population (*p* = 2.61 × 10^−4^). Likewise, the potential association of *LIPG* rs4939883 SNP and serum lipid profiles is contradictory. Kathiresan *et al*.^25^ observed that the rs4939883 SNP mutant allele was related to a significant decrease in plasma HDL‐C compared to subjects who carried the wild‐type allele in European populations (*p* = 1.6 × 10^−11^). The association of rs4939883 SNP and HDL‐C levels was replicated in another GWAS (*p* = 7 × 10^−5^).^26^ Nevertheless, a study investigating lipid‐related genetic loci in Caucasian women showed that the rs4939883 variant was highly associated with a lower level of ApoA1 (*p* = 1.9 × 10^−5^), although there was no association with HDL‐C.^27^ Teslovich *et al*.^28^ conducted a large GWAS including 95 loci related to lipid levels and have noted that the *LIPG* rs7241918 variant may contribute to variation in plasma HDL‐C and TC levels. More recently, Cui *et al*.^29^ reported that the rs7241918G allele was associated with higher serum HDL‐C concentrations in pregnant Chinese Han women. In the present study, we showed that the T allele carriers of *LIPG* rs2156552 SNP had lower levels of HDL‐C in both the Maonan and Han populations, and a lower ratio of ApoA1 to ApoB and ApoA1 levels in the Han population than the T allele non‐carriers. Subgroup analysis according to sex showed that the ratio of ApoA1 to ApoB only in Han males and the levels of HDL‐C and ApoA1 in Maonan females (but not in males) were different between AA and AT/TT genotypes (*p <* 0.0167). The minor allele T carriers of rs4939883 had higher levels of ApoB in the Han population and a lower serum HDL‐C level in the Maonan population than the minor allele non‐carriers (*p <* 0.0167). Subgroup analysis showed that there was no significant difference in genotypes and serum lipid parameters between males and females in both ethnic groups. In addition, we also identified that the G allele carriers of rs7241918 had a lower ratio of ApoA1 to ApoB in the Han population and a lower HDL‐C level in the Maonan population than the G allele non‐carriers (*p <* 0.0167). Subgroup analysis showed that serum ApoB levels and the ApoA1/ApoB ratio only in Han males were different between the GG and GT/TT genotypes (*p* < 0.0167–0.001). These results suggest that the *LIPG* SNPs can influence other serum lipid parameters except for HDL‐C in our study populations. However, the reason for these contradictions being related to the selected SNPs and serum lipid profiles between both ethnic groups is still unclear. It might be attributed to distinct genetic factors to some extent.

Although dyslipidemia is strongly associated with a genetic component, environmental factors, including lifestyle, physical inactivity, eating habits and being overweight, have been shown to reinforce lipid profile disorders. In the present study, we also showed that sex, weight, waist circumference, BMI, blood pressure, blood glucose levels, alcohol consumption and cigarette smoking were correlated with lipid profiles in both ethnic groups. The dietary patterns and lifestyle may be more disadvantageous for serum lipid profiles in the Maonan than in the Han population. The Maonan population comprises individuals of a traditional ethnic minority who make a living out of farming. Rice and corn are the most popular staple foods in the Maonan population. Many of them like to eat halfcooked food, pickle sour meat and animal offals, which contain abundant saturated fatty acids. A long‐term diet high in saturated fats has been associated with deleterious effects on serum lipid metabolism, especially because of their influence on serum TG, TC and LDL‐C levels.^40^

In the present study, we observed that the values for waist circumference and serum TG and ApoB were higher in the Maonan than in the Han population. Most of the local adult Maonan men like to drink and smoke, and consider it impolite not to give their guests wine. We also found that the the percentages of subjects who consumed alcohol and smoked cigarettes were higher in the Maonan than in the Han population. Excessive alcohol consumption and cigarette smoking have been shown to lead to dyslipidemia and related diseases.^41^ There is strong evidence for an adverse relationship between smoking and the metabolic and physiologic responses of different heart and blood vessels.^42^ Smoking initiates and promotes atherosclerosis by altering cardiac hemodynamics, causing dyslipidemia and the increased production of free oxygen radicals as a result of the oxidative stress of nicotine.^43^ Moderate alcohol consumption was shown to be causally related to a low risk of CAD by mainly increasing serum HDL‐C and ApoA1 concentrations; however, an excessive intake of alcohol has been shown to lead to hypertriglyceridemia.^44^ A previous meta‐analysis indicated that 30 g of alcohol daily was associated with a plasma TG increase of 5.69 mg/dl.^45^ Another study conducted in older Italian subjects (65–84 years old) found that alcohol intake increases serum LDL‐C levels.^46^ In summary, exposure to different environmental factors may further modify the effect of genetic variation on plasma lipid levels in the Maonan and Han populations.

There are several potential limitations to the present study. First, the sample size was relatively small compared to many GWAS and replication studies and further studies with larger sample sizes are needed to confirm our results. Second, serum lipid levels were examined only once, and so it hard to represent the long‐term status of lipid levels. Third, we were unable to alleviate the effect of diet and several environmental factors during the statistical analysis. Thus, *LIPG* expression in adipose tissue and serum lipid levels needs to be investigated in further studies.

## 5 CONCLUSIONS

Overall, the present study shows that the allelic (rs2156552, rs4939883 and rs7241918) and genotypic (rs2156552 and rs4939883) frequencies were different between the two ethnic groups. The association of *LIPG* polymorphisms and serum lipid levels was also different between the Maonan and Han populations, or between males and females. These results suggest that there may be a sex and/or racial/ethnic‐specific association of the *LIPG* SNPs and some serum lipid parameters in our study populations. These differences in the association of *LIPG* polymorphisms and serum lipid profiles between the two ethnic groups might partly result from different *LIPG*–enviromental interactions.

## CONFLICT OF INTEREST STATEMENT

The authors declare that they have no conflicts of interest.

## Supporting information


**Table S1** The sequences of forward and backward primers, restriction enzymes for genotyping of the LIPG SNPs.

**Figure S1** Map of Guangxi Huangjiang Maonan Autonomous County.
