# Supplementary material for: Association between the LIPG polymorphisms and serum lipid levels in the Maonan and Han populations
Source: J Gene Med. 2019 Feb 4;21(2-3):e3071. doi: 10.1002/jgm.3071 (PMC6590183; doi:10.1002/jgm.3071)
Supplement: Supplementary file 1 — Table S1 The sequences of forward and backward primers, restriction enzymes for genotyping of the LIPG SNPs. [file JGM-21-na-s001.docx]

**Association between the *LIPG* polymorphisms and serum lipid levels in the Maonan and Han populations**

Shuo Yang, Rui-Xing Yin*, Liu Miao, Qing-Hui Zhang, Yong-Gang Zhou, Jie-Wu

Department of Cardiology, Institute of Cardiovascular Diseases, The First Affiliated Hospital, Guangxi Medical University, Nanning 530021, Guangxi, People’s Republic of China

*LIPG* polymorphisms and serum lipid levels

* Correspondence: yinruixing@163.com

Department of Cardiology, Institute of Cardiovascular Diseases, the First Affiliated Hospital, Guangxi Medical University, 22 Shuangyong Road, Nanning 530021, Guangxi, People’s Republic of China

Shuo Yang, yangshuo1112@outlook.com

Rui-Xing Yin, yinruixing@163.com

Liu Miao, dr.miaoliu@qq.com

Qing-Hui Zhang, zhangqinghuixx@163.com

Yong-Gang Zhou, 3425874841@qq.com

Jie Wu, 516590618@qq.com

**Supplement Table 1** The sequences of forward and backward primers, restriction enzymes for genotyping of the *LIPG* SNPs

| SNP | Primer sequence | PCR  product | Restriciton enzyme | Restriciton fragment | Allele |
| --- | --- | --- | --- | --- | --- |
| rs2156552 | 5`-GACGCAACATGAAGGAGGTC-3` | 206 bp | Hpy188I | 206 | T |
|  | 3`-TATGTGCTGCCTACAACCCA-5` |  |  | 147+59 | A |
| rs4939883 | 5`-GAAGCTGTGCATCATGGGAG-3` | 427 bp | Hpy8I | 427 | T |
|  | 3`-CAAGGGGCAGAAGAAGAGGA-5` |  |  | 343+84 | C |
| rs7241918 | 5`-TCTTCTCCATCTCCCACCCT-3` | 512 bp | BseYI | 512 | T |
|  | 3`-ACATATACTGCTGGTGGGAA-5` |  |  | 416+96 | G |
