# Supplementary figures and images for: Association between the LIPG polymorphisms and serum lipid levels in the Maonan and Han populations
Source: J Gene Med. 2019 Feb 4;21(2-3):e3071. doi: 10.1002/jgm.3071 (PMC6590183; doi:10.1002/jgm.3071)

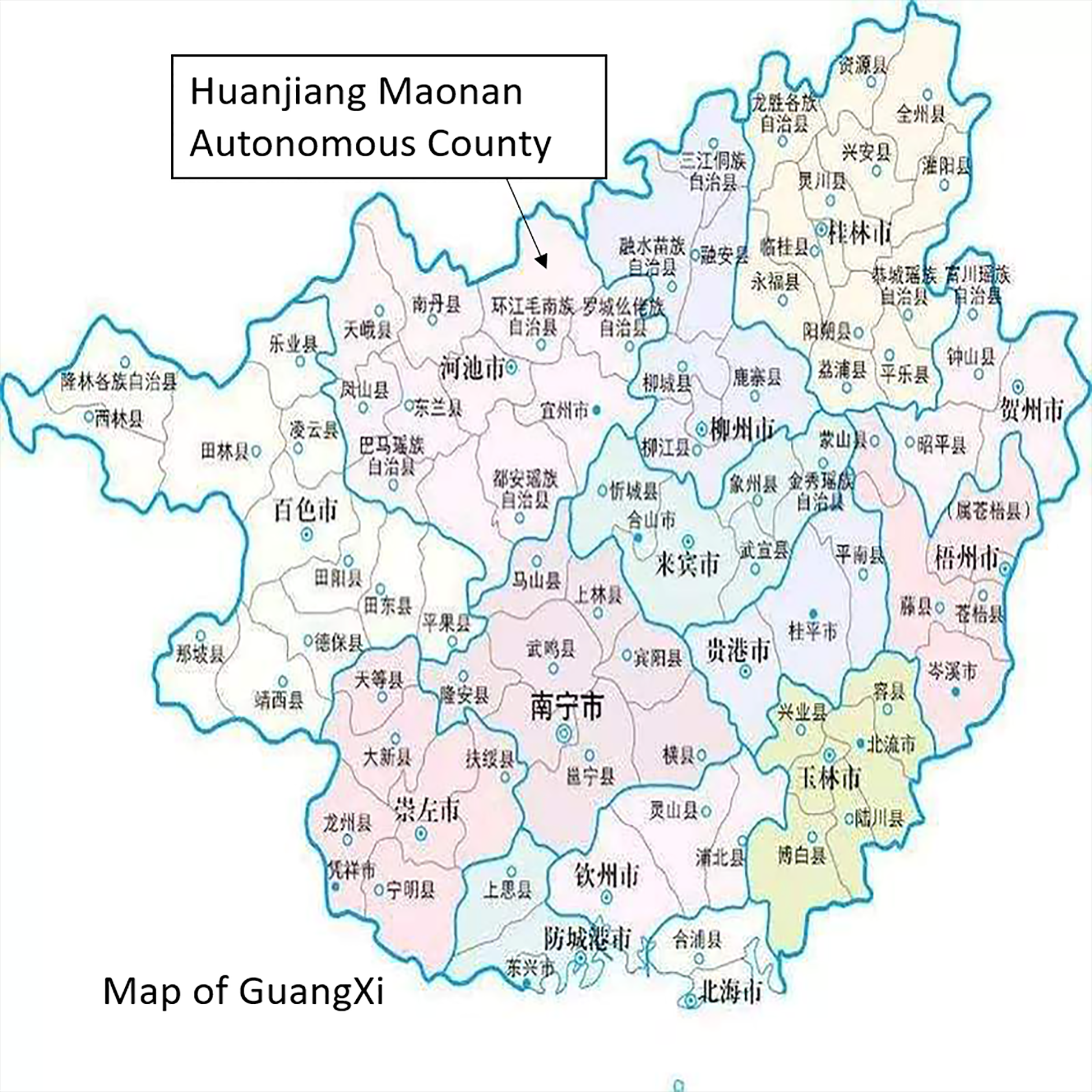

Supplement: Supplementary file 2 — Figure S1 Map of Guangxi Huangjiang Maonan Autonomous County. [file JGM-21-na-s002.tif]
